# Cochlear implants and 1.5 T MRI scans: the effect of diametrically bipolar magnets and screw fixation on pain

**DOI:** 10.1186/s40463-017-0252-9

**Published:** 2018-02-05

**Authors:** Ingo Todt, Grit Rademacher, Gloria Grupe, Andreas Stratmann, Arne Ernst, Sven Mutze, Philipp Mittmann

**Affiliations:** 1 Department of Otolaryngology, Head and Neck Surgery, Unfallkrankenhaus Berlin, Warenerstr.7, 12683 Berlin, Germany; 2 Department of Radiology, Unfallkrankenhaus Berlin, Berlin, Germany

**Keywords:** MRI, Cochlear implant, Complication, Pain, Diametrically bipolar magnet, Screw fixation

## Abstract

**Background:**

The probability that a patient will need an MRI scan at least once in a lifetime is high. However, MRI scanning in cochlear implantees is associated with side effects. Moreover, MRI scan-related artifacts, dislodging magnets, and pain are often the most frequent complications. The aim of this study was to evaluate the occurrence of pain in patients with cochlear implant systems using 1.5T MRI scans.

**Methods:**

In a prospective case study of 10 implantees, an MRI scan was performed and the degree of pain was evaluated by a visual analog scale. Scans were performed firstly with and depending on the degree of discomfort/pain, without a headband. Four of the cochlear implants contained a screw fixation. Six cochlear implants contained an internal diametrically bipolar magnet. MRI observations were performed with a 1.5 T scanner.

**Results:**

MRI scans were performed on all patients without causing any degree of pain, even without the use of a headband.

**Conclusion:**

Patients undergoing 1.5 T MRIs with devices including a diametrically bipolar magnet or a rigid implant screw fixation, experienced no pain, even without headbands.

## Background

Cochlear implantation is the treatment of choice for patients with severe hearing loss, and those who have profound hearing loss (deafness affecting one or both ears). So far, about 400,000 patients have been provided with a cochlear implant.

MRI observations in cochlear implantees are a longterm problem. Because the fixation of the implant audio processor is magnet–based, MRI scans need special modifications to circumvent possible inaccuracy. On the other hand, previous trials with magnet-free implant systems did not lead to significant acceptance [1].

The internal cochlear implant magnet leads to a number of testing difficulties. The magnet generates significant artifacts, leading to the inability to assess specific ipsilateral structures [2]. Magnet dislocations can occur, cause pain, and act as a source of infections [3]. Depending on the sequencing used and the position of the implant, the visibility of the scan can be modified to allow an assessment of the cochlea and the internal auditory canal [4, 5]. With specific headbands, the number of magnet dislocations described are rare [6]. However, the occurrence of pain during the scan is a frequent complication even in cases where a dislocation did not occur [6, 7]. Often MRI scans cannot be performed because of the related pain [7].

Concerning how specific devices measure rates of pain, Grupe et al. [6] described an overall pain/ discomfort rate of 70% including Advanced Bionics 90 K, 90 K Advance devices, Cochlear 512, 422, 24 RCA and MEDEL Concerto systems.

Kim observed pain/discomfort in 7 out of 19 cases without performed general anesthesia [7] for the scanning. Causing devices were Nucleus 24 RCA, 22 M, Advanced Bionics 90 k and CII. Crane [8] described a general mild sedation protocol, but in two out of 22 scans (Nucleus, 90 k) pain-related problems occurred. Carlson found in two out of 34 scans the occurrence of pain, which did not allow a regular completion of the scan [9] with Nucleus 24 devices.

Therefore, the use of an MRI in a cochlear implantee warrants special consideration. Because all of these side effects are well-known, manufacturers (Cochlear Corp., Sydney, Australia; Advanced Bionics, Stäfa, Switzerland; Medel, Innsbruck, Austria; Oticon, Valaudaris, France) recommend head bandages for safety reasons for 1.5T (Cochlear 24, 422, 512, 522, 532; Advanced Bionics 90 k, 90 k Advance, Ultra; MEDEL Concerto, Synchrony; Oticon ZTI), or magnet removal at 3T (Cochlear 24, 422, 512, 522, 532; Oticon ZTI) or headbands for 3T (Medel Synchro-ny). Generally different from other systems are two MRI-relevant device specifications. One option is to use screws to anchor the implant (Neuro ZTI, Oticon, Valaudaris, France), and the other is to incorporate a diametrically bipolar magnet (Synchrony, Medel, Innsbruck, Austria) to decrease the force on the implant and therefore prevent magnet dislocations, demagnetization and pain.

The aim of this study was to evaluate the occurrence of pain in patients with cochlear implant systems using 1.5T MRI scans.

## Methods

The study was approved by the institutional review board of the Unfallkrankenhaus Berlin, Germany (IRB-ukb-HNO-2016/01). Patients gave their written informed consent for the use of their clinical records in this prospective study.

In this prospective case study, from 2014 through 2016, 10 patients under-went a 1.5T MRI observation provided in a tertial referral center with varying cochlear implant types. Six patients were implanted with a Medel Synchrony implant (Medel, Innsbruck, Austria) and a bipolar magnet. The implant was intraoperatively anchored with a absorbable suture. An implant bed was drilled for these cases. Four patients were implanted with an Oticon Neuro ZTI implant (Oticon, Vallauris, France). The implant was only fixed with two selftaping screws. An implant bed was not drilled.

All examinations were performed in a 1.5 Tesla MR imaging unit (Ingenia, Philips Medical Systems, Best, NL) using an 8-channel array head coil. All patients underwent two MRI scans. First the patient was introduced into the MRI scanner with a headband. The headband consists of a tight self-taping wrap with a hard plain piece to prevent dislodging of the magnet. After the evaluation of pain and inspection of the implant area, the patient was introduced into the scanner without a headband a second time.

The evaluation of pain was performed with a visual analog scale (VAS) scoring from 0 to 10. Zero indicated the non-occurrence of pain and discomfort. Ten indicated a pain-related interruption during the scan. The questionnaire was used directly after the first and directly after the second scan. Magnet strength was subjectively evaluated by the attraction force of the antenna coil. Magnet displacement was evaluated by the digital control of the implant magnet area.

Scanning parameters:

TSE T2 2 D: TR: 3300 ms, TE 120 ms, slice thickness 1.5 mm, reconstruction resolution of 0.55 × 0.55 × 1.5 mm, F0 V 120  ×  120. 12 slices.

TSE T1 2 D: TR: 550 ms, TE 20 ms, slice thickness 3 mm, reconstruction resolution of 0.23 × 0.23 × 3 mm, F0 V 120  ×  120. 20 slices, matrix size: 400  ×  318.

## Results

In all patients, two MRI scans at 1.5T were performed without complications, in terms of magnet dislocation and pain. One scan with the headband and one scan without the headband were performed. In all cases, a headband was not necessary to prevent pain or magnet dislocation. The mean VAS with and without head-band, in terms of pain, was 0. The evaluation was performed directly after the scan. Individual data are given (Table 1). A change of the magnet strength or polarization was not observed for either implant.

**Table 1:** Patients individual MRI data

Name	sex	age	implant	VAS for MRI with headband	VAS for MRI without headband
Pat.1	f	45	MEDEL Synchrony	0	0
Pat.2	m	56	MEDEL Synchrony	0	0
Pat.3	f	72	MEDEL Synchrony	0	0
Pat.4	f	43	MEDEL Synchrony	0	0
Pat.5	f	43	MEDEL Synchrony	0	0
Pat.6	f	35	MEDEL Synchrony	0	0
Pat.7	f	65	Oticon ZTI	0	0
Pat.8	m	67	Oticon ZTI	0	0
Pat.9	f	45	Oticon ZTI	0	0
Pat.10	f	70	Oticon ZTI	0	0

## Discussion

MRI observations are frequently necessary in cochlear implantees for head-related reasons (exclusion of tumor, unclear vertigo, exclusion of infarction) or non-head-related reasons. The probability for an MRI once in a lifetime has been described to be 50–75% [10]. 1.5T MRI scans for cochlear implantees are associated with artifacts [8] and magnet dislocations [5], and, as a most frequent topic, pain [6]. At 3T additional demagnetization has been described [2].

While manufacturers recommend a headband during the MRI scan to prevent pain and magnet dislocation, it has been shown to be ineffective [6] in many cases. Pain is often the reason not to scan. The complication rate of MRI scans due to pain or discomfort is between 70% [6], 2 out of 22 [8], 7 out of 19 [7] and 2 non-tolerated cases out of 34 cases of MRI scans experience pain or discomfort [9]. All studies, to date, have used implants that contain magnets in silicon pockets or rigid incorporated magnets with a non-fixed implant body. Out of the experience with the two observed implant specifications in terms of pain, it can be assumed that the movement of the implant magnet out of the silicon pocket (Nucleus 24 to 512, Advanced Bionics 90 k) or the implant itself (Advanced Bionics CII, Medel Concerto) causes pain during scanning.

It can be assumed that the mechanism of pain occurrence is related to the magnet movement inside the MRI magnet field which results in a hard-to-contact signal between magnet,periostium and inner skin layer.

Clinically the effectiveness of a diametrically bipolar magnet and screwing fixed implant modification to prevent this complication is unknown and therefore the first description of this topic.

Medel Synchrony implant includes a diametrically bipolar magnet, which directs itself in the magnetic field of an MRI scanner. This direction was assumed to prevent demagnetization at 3T. The effect on pain prevention is so far unclear. We were able to show that the implant magnet configuration even prevents the occurrence of pain. A magnet dislocation was not observed.

The second implant (Oticon Neuro ZTI) includes a rigid implant corpus with a magnet to screw in and out of the corpus. The implant itself is fixed with screws on the skull. This anchor seems to prevent a torsion of implant and magnet to press on the periost and skin. Magnet dislocation could not be observed.

The prevention of pain and magnet dislocation is of high importance because these two factors are the most frequent and relevant in the clinical routine.

The ability to perform an MRI scan without pain or the risk of magnet dislocation offers the clinician and the radiologist the field to perform 1,5T MRI scans routinely with only some limitations (e.g., artifact relevant limitations, Tesla strength limitations, demagnetization for ZTI Implant at 3 T).

## Conclusion

Patients undergoing 1.5 T MRIs with devices including a diametrically bipolar magnet or a rigid implant screw fixation have no pain, even without headbands.

*All procedures performed in studies involving human participants were in accordance with the ethical standards of the institutional and/or national research committee and with the 1964 Helsinki declaration and its later amendments or comparable ethical standards.*

## Abbreviations

CI: Cochlear implant
MRI: Magnet resonance imaging
T: Tesla
VAS: Visual analog scale
